# Draft Whole-Genome Sequence of *Sphingobium* sp. Strain PNB, a Versatile Polycyclic Aromatic Hydrocarbon-Degrading Bacterium

**DOI:** 10.1128/MRA.00920-21

**Published:** 2021-12-02

**Authors:** Madhumita Roy, Tapan K. Dutta

**Affiliations:** a Department of Microbiology, Bose Institute, Kolkata, India; University of Southern California

## Abstract

*Sphingobium* sp. strain PNB can completely degrade phenanthrene, naphthalene, and biphenyl as the sole carbon and energy source. The strain is also capable of cometabolizing benzo[a]pyrene, pyrene, acenaphthene, fluoranthene, etc. Here, we report the 5.69-Mb assembly and annotation of the genome sequence of strain PNB, obtained using Illumina sequencing.

## ANNOUNCEMENT

*Sphingobium* sp. strain PNB is a Gram-negative, rod-shaped, chemoheterotrophic, strictly aerobic bacterium that forms yellow colonies. It was isolated from a municipal waste-contaminated soil sample from Dhapa, Kolkata, India (22.5373°N, 88.4334°E), and selected due to its versatile capability to completely degrade various aromatics, like phenanthrene, naphthalene, and biphenyl, as the sole source of carbon and energy (1). Strain PNB can also partially utilize anthracene as the sole carbon source, producing 3-hydroxy-2-naphthoic acid as the dead-end metabolite, and is able to cometabolize a number of high-molecular-weight polycyclic aromatic hydrocarbons (PAHs) in the presence of phenanthrene (2–4). Thus, due to its versatile catabolic potential regarding various PAHs and other aromatics, including central metabolites, information about the genome sequence of the strain would be helpful in future for directed strain improvement by targeted genetic engineering for biotechnological applications, including PAH bioremediation.

For molecular characterization, *Sphingobium* sp. strain PNB, originally isolated from soil and maintained in the lab, was grown in lysogeny broth (LB) medium (5) at 29°C for 24 h, and high-quality DNA was extracted using the GeneJET genomic DNA purification kit (Thermo Fisher Scientific, MA, USA). The concentration and quality of the extracted DNA were determined using the PHERAstar FSX multimode microplate reader (BMG Labtech). The genomic DNA was subjected to sequencing via Illumina (MiSeq platform) with paired-end technology (2 × 250 bp), and barcode strategies were applied according to the manufacturer’s instructions (Illumina, USA). A sequencing library was prepared using the Nextera XT DNA library preparation kit (Illumina). Both paired-end sequencing read files were separately subjected to the FastQC v0.115 tool, and the necessary trimming was executed using the FASTX-Toolkit v0.0.14 (http://hannonlab.cshl.edu/fastx_toolkit/). The most suitable kmer size was estimated in the processed reads of the *Sphingobium* genome by applying KmerGenie v1.7051 software (6). In this analysis, the best kmer size obtained was 85. The SPAdes v3.15.0 assembler was utilized for *de novo* assembly using the good-quality processed reads (7). This analysis was performed using the “careful” option, which is recommended for mismatch correction. A total of 332 scaffolds were constructed in the *de novo* assembly. After the removal of some small scaffolds (<500 bp), a total of 242 scaffolds were obtained. The exact *N*_50_ value was 49,683 bp, and the sequencing coverage was 10×. In all, 5,696,236 bp (∼5.7 Mb) of assembled consensus sequences were obtained, with a GC content of 64.11%. Functional annotation of the genome was performed using the NCBI Prokaryotic Genome Annotation Pipeline (PGAP) (http://www.ncbi.nlm.nih.gov/genomes/static/Pipeline.html) (8). The total number of genes and coding DNA sequences (CDS) annotated were 5,553 and 5,491, respectively. In the above analyses, default parameters were used for all software unless otherwise specified.

The genome harbors pathways for the degradation of PAHs/biphenyl, catechol (*ortho*- and *meta*-cleavage), homogentisate, salicylate, phenol (containing multicomponent hydroxylase), nonoxidative decarboxylase-mediated aromatic acid, etc. In addition, chromium, copper, and arsenic resistance genes were found in the genome, suggesting the suitability of the strain for the bioremediation of diverse PAH-contaminated sites.

### Data availability.

The raw reads are available under NCBI BioProject accession number PRJNA757604. The raw reads are available under SRA accession number SRX11971035. The *Sphingobium* sp. strain PNB whole-genome shotgun sequencing project has been deposited in DDBJ/ENA/GenBank under accession number JAINEZ000000000. The version described in this paper is JAINEZ010000000.
